# Effectiveness of an Integrated Nutrition Rehabilitation on Growth and Development of Children under Five Post 2018 Earthquake in East Lombok, Indonesia

**DOI:** 10.3390/ijerph19052814

**Published:** 2022-02-28

**Authors:** Umi Fahmida, Ahmad Thohir Hidayat, Anak Agung Sagung Indriani Oka, Dini Suciyanti, Pathurrahman Pathurrahman, Grace Wangge

**Affiliations:** 1 Southeast Asian Ministers of Education Organization Regional Center for Food and Nutrition (SEAMEO RECFON), Pusat Kajian Gizi Regional Universitas Indonesia, Jakarta 10430, Indonesia; mrthoin@gmail.com (A.T.H.); indrianioka@gmail.com (A.A.S.I.O.); dinisuciyanti@gmail.com (D.S.); gwangge@gmail.com (G.W.); 2 Early Childhood Care, Nutrition and Education (ECCNE) Working Group, Southeast Asian Ministers of Education Organization Regional Center for Food and Nutrition (SEAMEO RECFON), Jakarta 10430, Indonesia; 3 East Lombok District Health Office, East Lombok, Selong 83611, Indonesia; rpathur211@gmail.com

**Keywords:** child development, child growth, optimized complementary feeding recommendations, early childhood development, early childhood education center, nutrition rehabilitation, optimized food-based recommendation, post-disaster, psychosocial care, children under five

## Abstract

Background: In August 2018 Lombok Island in Indonesia was hit by a 7 Richter scale earthquake. This study aimed to assess the effectiveness of comprehensive nutrition disaster rehabilitation, based on the holistic integrated early child development concept, on the growth and development of children under five. Methods: A community-based intervention was performed in the East Lombok district; four villages in two sub-districts were randomly allocated into intervention or control groups. Mothers of 6–49-month-old children in the intervention group (*n* = 240) attended parenting classes (twice weekly) and received shredded fish/liver/anchovy and optimized complementary feeding/food-based recommendations, developed using linear programming. Health staff from the public health center and teachers from early childhood education (ECE) centers delivered parenting sessions on health–nutrition and care–education. The control group (*n* = 240) received existing health services. Indicators measured at baseline and the end line point were weight, length/height, hemoglobin, feeding practices, psychosocial care (HOME) and maternal stress (SRQ). At the end line point, child development was assessed using BSID-III. Results: At the end line point, maternal stress and child morbidity (cough) were lower and dietary diversity (+1) in 6–23-month-old children, and weight-for-age Z-score (+0.26) and social emotional score (+10 points) in ≥24-month-old children were higher in the intervention group. Conclusions: The nutrition rehabilitation intervention delivered through ECE centers has a positive effect on the growth and development of children under five in post-disaster conditions.

## 1. Introduction

Indonesia is situated in the Ring of Fire, which is prone to natural disasters. In August 2018 Lombok Island in Indonesia was hit by a 7 Richter scale earthquake. Disruptions of access to clean water, food and health services and maternal stress caused by the disaster could have had negative consequences for the nutritional status of vulnerable groups, especially young children.

The 2011 Edition of the Sphere Handbook Humanitarian Charter and Minimum Standards in Humanitarian Response emphasizes the importance of nutritional assessments beyond anthropometric surveys to assess the extent of and underlying causes of undernutrition. In particular, infant and young child feeding practices, micronutrient deficiencies and links with other sector assessments, particularly food security and livelihood, water and sanitation and health are recommended [1], which should be conducted soon after the disaster and not later than 3–6 months post-disaster [2]. A specific rehabilitation and mitigation intervention needs to be conducted to prevent a nutritional status deterioration in children post-disaster. The 2016 Lancet Early Childhood Development (ECD) Series highlights the importance of early childhood development and identified interventions that have significant benefits for early childhood development. These interventions include parenting programs, maternal mental health and wellbeing, social protection, water, sanitation and hygiene. The series also emphasizes the importance of multi-sectoral interventions starting with health, which can have a wide reach for families and young children through health and nutrition [3]. The holistic integrated ECD concept (also known in Indonesia as *Pengembangan Anak Usia Dini Holistik Integratif* or PAUD HI) has been promoted by the Ministry of Education and Culture in Indonesia [4]. However, translation of this, especially in the context of the rehabilitation and mitigation of child growth and development in disaster or emergency situations has not yet been reported.

To prevent negative consequence of emergency situations on child growth and development, we developed integrated nutrition rehabilitation targeted at children under five and their caregivers, which translated the holistic integrated ECD concept. Early child education (ECE) centers are existing community resources that are available in every village in Indonesia; therefore, they have potential to be used as a channel for delivering nutrition rehabilitation post-disaster. Utilizing the ECE centers together with the public health service also supports multi-sectoral interventions, i.e., the collaboration of both health and non-health sectors. This study aimed to assess the effectiveness of comprehensive nutrition rehabilitation intervention based on holistic integrated ECD in reducing maternal stress and child morbidity, improving psychosocial care, dietary intake and eventually the nutritional status and developmental outcomes of children under five whose families had just been affected by the 2018 Lombok earthquake.

## 2. Materials and Methods

### 2.1. Study Design and Study Population

The study was community-based intervention trial (community-based cluster randomized controlled trial, cRCT) conducted between February and September 2019. The baseline assessment was conducted in December 2018, i.e., four months after the earthquake. Subjects were children aged 6–49 months old. Mothers of the children in the intervention group (*n* = 240) attended parenting classes twice a week to receive shredded fish, liver or anchovy as nutrient-dense foods, which can fill the gap of problem nutrients (iron, zinc, calcium), as well as optimized food-based recommendations developed using linear programming approach. Health staff from public health center and teachers from early childhood education (ECE) centers delivered parenting sessions related to health–nutrition and education–care.

The inclusion criteria were children aged 6–49 months old at baseline, apparently healthy and had been staying in the area for at least the previous six months. The exclusion criterion was mother or caregiver was not present during the baseline assessment.

### 2.2. Sample Size and Sampling

The minimum sample size (*n* = 213/group) was calculated based on α = 5% and power = 90%, difference in weight gain of 0.5 g/kg per day with 1.5 design effect following the Standardized Monitoring and Assessment of Relief and Transitions (SMART) guidelines [5].

Two sub-districts (*Sambelia* and *Pringgabaya*) were selected. *Sambelia* (0–930 m above sea level) was located in the center of the earthquake and was mostly hit by the earthquake, whereas *Pringgabaya* (0–250 m above sea level) was densely populated sub-district and therefore a lot of people were affected by the earthquake. From each sub-district, two villages were selected and were randomly assigned into either intervention or control group. Within each village, six ECE centers were selected (total of 24 ECE centers).

### 2.3. Intervention

Based on previous study [6], the typical problem nutrients of young children in East Lombok were iron, zinc and calcium. The shredded liver, fish and anchovy were previously reported to have positive effects in improving intakes and nutrient densities of these nutrients as well as other nutrients. Therefore, in this nutrition rehabilitation we delivered shredded liver, fish and anchovy twice weekly in the ECE centers. The shredded liver, fish and anchovy were prepared locally and involved mothers and voluntary health cadres with supervision from study team. During twice weekly sessions, nutritionists from public health center (*Puskesmas*) delivered messages on optimized complementary feeding and food-based recommendations (CFR/FBR), food safety and integrated management of child illness. In addition, ECE teachers delivered sessions related to parenting, child welfare and protection and psychosocial stimulation. Nutritionists and ECE teachers had been trained on the modules on infants and young child feeding with optimized CFR/FBR and playing with young children. Both modules had been designed based on local conditions and availability of foods and resources for child stimulation available in the area. Health staff from *Puskesmas* monitored monthly using checklist form to assess compliance of mothers in feeding their children the shredded liver, fish and anchovy as well as to monitor the overall implementation of the sessions.

### 2.4. Ethics and Informed Consent

The Ethics Committee of the Faculty of Medicine, University of Indonesia (Permit number 0985/UN2.F1/ETIK/2018) granted ethical approval for the study. Permission was obtained from the local government (district levels) and local health authority before assessment started. The involvement of respondents in this survey was voluntary. The interview was held with the respondents’ agreement only after they had received and signed the informed consent form. The study was registered at clinicaltrials.gov (registration number NCT03509155).

### 2.5. Data Collection

Interviews and measurements were conducted by trained enumerators with supervision from field supervisors who monitored the data quality. Structured interviews were conducted to assess socio-demography condition of the family, environment condition, infant and young child feeding (IYCF) practices [7], psychosocial care using Home Observation for the Measurement of Environment (HOME) [8] and maternal stress using Self Rating Questionnaire (SRQ) [9]. Dietary intake data were collected using a 4-pass 24-h dietary recall (24-HR) at baseline and end line [10]. Portion sizes were estimated by weighing if foods were available to be weighed, or estimated using food photographs. Food frequency questionnaire was used to assess the consumption of selected food groups and subgroups based on the CFR/FBR in the previous week prior to baseline and end line assessments. Compliance to consuming the nutrient-dense foods (shredded liver, fish and anchovy) was recorded by checklist conducted monthly by health staff from *Puskesmas*.

Weight, recumbent length (children < 24 months old), height (children 24–59 months old) were measured in duplicate by trained enumerators. Weight was measured using SECA 876 to the nearest 0.1 kg and length/height using shorrboard to the nearest 0.1 cm. The anthropometrist took a third measurement if the difference between the first two measurements exceeded a predetermined allowable limit (0.1 kg for weight and 0.7 cm for length/height) [11]. Hemoglobin of children was assessed by Hemocue using finger-prick blood. Anemia was defined as hemoglobin of less than 11.0 g/dL. Developmental outcomes were assessed at end line point by trained psychologists using BSID-III [12].

### 2.6. Data Analysis

Data analysis was performed using IBM SPSS Statistics 21. Differences between groups were analyzed using Chi-square for binomial categories, unpaired *t*-test for continuous variable (if normally distributed) and Mann–Whitney (if not normally distributed). Differences between intervention and control groups were tested using ANCOVA (for continuous variables) or logistics regression (for binomial variables) with variables found to be different at baseline used as covariates.

## 3. Results

All subjects who were brought to the ECE centers came with their caregivers, therefore no subject was excluded. Table 1 presents the household-level characteristics of the subjects. Most fathers (97%) and mothers (98%) were of *Sasaknese* ethnicity (local of Lombok) and there was a comparable education level of the fathers between the two groups. There was, however, a difference between the intervention and control area in terms of the mother’s education and type of family, i.e., more mothers in the control group did not reach the minimal education of nine years (junior high) and were from nuclear, instead of extended, families. There were more boys in the intervention than the control group (54.7% and 45.3%, respectively, *p* = 0.041); however, child age was not significantly different between groups, i.e., medians (min–max) were 30 (10–42) months and 27 (9–38) months in the intervention and control groups, respectively.

Immediately after the earthquake, most households stayed at the refugee camps and 5% still stayed at the camp during the baseline. One-third of the families experienced difficulties in accessing foods and basic needs, and more in the control group experienced difficulty to access health services and damaged houses. Although fewer than 5% experienced fatalities and injured family members, most (>90%) mothers reported feeling traumatized by the earthquake, which lasted for some time after the major earthquake happened. Nevertheless, most mothers of <24-month-old children had continued breastfeeding their children.

Compliance to the shredded fish, liver and anchovy were 99%, 91% and 91%, respectively. Among children less than 24 mo (month), the dietary diversity score (DDS) was comparable at baseline but was significantly higher in the intervention group at the end line assessment. In the intervention group, there was a higher consumption (serves/week) of animal source foods, particularly of liver, poultry, eggs (*p* < 0.05) and seafoods (*p* < 0.1), Table 2.

Although diarrhea, runny noses and coughs were higher in the intervention group at baseline, the prevalence decreased and was no longer different than the control group at the end line, although coughs were lower than the control group at the end line assessment. All illnesses decreased in the intervention group; on the contrary, there was an increase in coughs and runny noses in the control group (Table 3).

At baseline, there was no difference between the two groups in hemoglobin, weight-for-age and weight-for-length or height Z-scores both among children aged 6–23 months and ≥24 months. At the end line assessment, among the ≥24-month-old children height-for-age and weight-for-age Z-scores were higher in the intervention than the control group. (Table 4).

At baseline there were higher SRQ scores in the intervention group, which showed higher maternal stress. At the end line assessment the score was reduced in the intervention group and it was no longer different than the control group. The proportions of mothers having depression (SRQ score ≥ 6) reduced significantly in the intervention group (61% at baseline, 43% at end line, *p* < 0.001, McNemar test) but not in the control group (43% at baseline, 40% at end line, *p* = 0.272, McNemar test). Although not statistically significant, the median HOME was 1 point higher in the intervention group at end line. The developmental scores assessed at end line showed 10 point higher social emotional scores (95 vs. 85) in ≥24-month-old children in the intervention group (Table 5).

## 4. Discussion

Results showed that the integrated intervention to optimize child growth and development post-earthquake in East Lombok can be conducted with active participation from parents, health staff and ECE teachers. After the 6 months of intervention, maternal stress and morbidity was lower in the intervention group. Among the <24-month-old children, dietary diversity was also higher in the intervention group. A positive effect on the WAZ and social emotional score was observed in ≥24-month-old children. It is to be noted that this is the first study reporting the effect of a community-based nutrition rehabilitation post-disaster on both growth and developmental outcomes of children.

Our baseline data showed higher prevalences of diarrhea and upper respiratory infections (URI) than those reported in the national basic health survey (*Riskesdas*) 2018, which represent a non-disaster situation. Based on *Riskesdas* 2018 the prevalences of diarrhea and URI were 10.2% and 11.7% (West Nusa Tenggara Province) and 8.0% and 9.3% (national), respectively [13]. The prevalence of diarrhea at baseline was two times the national figure but returned to close to the national figure at the end line assessment. The prevalence of upper respiratory infections (cough) was high at baseline and remained high at the end line point, and even increased in the control group. This finding was concurrent with increased intakes of animal source foods in this study. A previous study and review showed that intervention with animal protein did not significantly reduce morbidity [14,15]. The studies reviewed in the Cochrane Review, while reporting mostly the LMIC settings, did not target the caregivers nor target developmental outcomes; therefore, our finding provides additional information that has not been previously reported.

In this study, we did not find significant difference between groups in terms of hemoglobin or weight-for-length (WLZ) or weight-for-height (WHZ) Z-scores. There was a lack of evidence of the effect of nutrition intervention post-disaster on child growth. A study in Niger on the effect of unconditional cash transfers (CT) during a nutritional emergency showed a pre–post change in WLZ or WHZ of 0.20; however, the study did not include a control group, therefore, it was not possible to attribute the effect to the CT program [16]. A more recent publication of an emergency CT program promoted weight gain and reduced acute malnutrition risk among children 6–24 months old during the food crisis in Niger [17]. Compared to our study, in the Niger study only children <24 months old were included, and their mean WHZ was much lower (−1.5 vs. −0.85) and median dietary diversity score was also lower (3 vs. 5), which explained the greater response on weight gain and improved DDS. In our study, mean changes in WLZ or WHZ showed a decrease in children <24 months old (−0.20) but an increase in ≥24-month-old children (+0.26). This finding suggests that the youngest children (<24 months old) especially were more vulnerable during the post-disaster condition; therefore, more attention should be given for this group, particularly in providing adequate energy and nutrient intakes through complementary feeding.

The intervention also improved the dietary diversity score, particularly the intake of animal source foods, which are sources of the identified problem nutrients in the area, i.e., iron, zinc and calcium. Liver, fish and anchovy were identified as the nutrient-dense foods locally available that could increase the intakes of these problem nutrients [6]. To our knowledge, our study is the first study that promoted food-based recommendations developed using a linear programming approach in the emergency setting.

Child development has been associated with parental factors including parent–child relationships [18]. The intervention in this study included sessions to improve parents’ awareness of their parenting style and to guide them to provide psychosocial stimulation for their children according to their age. The important effect of the intervention in our study is in reducing maternal stress. Maternal mental health has been shown to have a large impact on several domains of development of 3–24-month-old children, i.e., gross and fine motoric as well as personal–social subscales [19].

Our study showed the effect of integrated holistic ECD, which involves not only the health sector but also the education sector. The provision of food supplements for children were prepared and distributed by the local ECE teachers who also served as part of the impacted community. Our finding supports previous studies that identified the important role of local community resilience and that effective use and coordination of community resources can help community recovery post-disaster [20,21]. The study included a control group and there was low dropout throughout the 6 month intervention. The intervention also included an integrated component aimed at both the growth and developmental outcomes of the children and assessed the comprehensive indicators related to holistic integrated ECD, i.e., dietary intake, morbidity, maternal stress, psychosocial care, nutritional status and developmental outcomes.

The limitations of this study are first, the intervention started sometime after the disaster, therefore may not reflect the conditions immediately after the disaster, and other assistance may have contributed to the condition. Second, baseline characteristics between the intervention and control groups were not comparable in family type, water source and difficulty in access to health service, which were significantly associated with the study outcomes (wasting, cough, runny nose). Finally, we could only assess the developmental outcomes at the end line point.

## 5. Conclusions

In summary, the integrated nutrition rehabilitation intervention had benefits in reducing maternal stress and child morbidity and in improving dietary diversity (in <24-month-old children), weight-for-age and development (in ≥24-month-old children) of the children in post-disaster conditions. ECE centers have the potential to be used as a community-based channel to deliver holistic integrated ECD involving a health, education and inter-sectoral approach to protecting child growth and development in post-disaster conditions. Further scale up of this holistic integrated ECD approach in a programmatic setting is recommended, particularly in countries prone to natural disasters and emergency situations.

## Figures and Tables

**Table 1:** Socio-demography characteristics of study subjects, by treatment group.

Characteristics	Intervention (N = 240)		Comparison (N = 240)		*p*-Value ^1^
	*n*	%	*n*	%	
Type of family: nuclear	147	61.3	178	74.2	0.002
Father’s education					0.640
- No schooling	24	10.2	27	11.4	
- Primary	61	25.5	71	29.7	
- Junior high	61	25.5	64	26.5	
- Senior high	72	30.1	57	23.7	
- Diploma/higher	21	8.8	21	8.7	
Mother’s education					0.008
- No schooling	10	4.3	30	12.5	
- Primary	64	26.7	70	29.3	
- Junior high	77	31.9	77	31.9	
- Senior high	61	25.4	41	17.2	
- Diploma/higher	28	11.6	22	9.1	
Stay at refugee camp after the earthquake	181	75.4	192	80.0	0.228
Still stay at the refugee camp at baseline	11	4.6	12	5.0	0.482
House broken down after the earthquake	86	35.2	140	58.3	<0.001
Public facility around is unusable	46	19.2	50	20.8	0.648
There are fatalities from family members	3	1.3	8	3.3	0.127
There are injured family members	11	4.6	7	2.9	0.337
Feeling traumatic	221	92.1	217	90.4	0.518
Difficulties in accessing food and basic needs	66	27.5	73	30.4	0.481
Difficulties in accessing health service, education and place of worship	11	4.6	44	18.3	<0.001
Breastfeed child at refugee camp ^2^	70	95.9	66	75.0	0.484
Stop breastfeed child because of the earthquake ^2^	2	2.7	4	4.5	0.581
Toilet facility					0.106
- Private toilet	193	80.4	172	71.7	
- Public toilet	19	7.9	29	12.1	
- River/open space	27	11.3	35	14.6	
- Others	1	0.4	4	1.7	
Source of drinking water					<0.001
- Bottled water	3	1.3	9	3.8	
- Piped water	75	31.3	39	16.3	
- Well	151	62.9	115	47.9	
- Spring	6	2.5	74	30.8	
- Others	5	2.1	3	1.3	

^1^ Chi-square to test for independence to assess any significant difference between treatment groups. ^2^ in children < 24 months old only (intervention *n* = 73; comparison *n* = 88). *p* < 0.05 indicates statistical significance.

**Table 2:** Dietary diversity and frequency of consumption of food groups, subgroups and food items, by treatment group ^1^.

Variable	Intervention (N= 200)	Control (N= 215)	*p*-Value ^2^
**Dietary diversity score ^3^**			
Baseline	5 (3–7.0)	5 (3–6)	0.065
End line	7 (6–9)	6 (5–8)	0.003
**Animal protein**			
Baseline	7 (3–13)	7 (5–10)	0.559
End line	14 (9–18)	12 (8–17)	0.060
**Eggs**			
Baseline	2 (1–4)	2 (1–4)	0.994
End line	3 (2–7)	3 (2–6)	0.021
**Liver**			
Baseline	0 (0–0)	0 (0–0)	0.380
End line	0 (0–1)	0 (0–1)	<0.001
**Poultry**			
Baseline	1 (0–2)	1 (0–2)	0.396
End line	2 (1–3)	1 (0–2)	0.010
**Fish**			
Baseline	2 (0–5)	2 (1–4)	0.121
End line	3.5 (2–5)	4 (1–6)	0.806
**Small fish with bone**			
Baseline	0 (0–0.75)	0 (0–0)	0.065
End line	1.5 (0–3)	1 (0–3)	0.663
**Red meats**			
Baseline	0 (0–0)	0 (0–0)	0.533
End line	0 (0–0)	0 (0–0)	0.343
**Seafoods**			
Baseline	0 (0–0)	0 (0–0)	0.294
End line	0 (0–1)	0 (0–1)	0.056
**Plant protein**			
Baseline	4 (1–1.75)	4 (2–7)	0.934
End line	6 (4–9)	5 (3–8)	0.208
**Tahu/Tempe**			
Baseline	3 (1–7)	3 (2–5)	0.799
End line	4 (3–7)	4 (2–7)	0.253
**Legumes**			
Baseline	0 (0–0)	1 (0–1)	0.007
End line	1 (0–2)	1 (0–2)	0.499
**Vegetables**			
Baseline	5 (3–7)	7 (4–8)	0.014
End line	7 (5–14)	7 (4–10)	0.040
**Dark green leafy vegetables (DGLV)**			
Baseline	3 (2–7)	5 (3–7)	0.016
End line	4.5 (3–7)	4 (3–7)	0.158
**Other vegetables**			
Baseline	0 (0–2)	1 (0–2)	0.008
End line	1 (0–2)	0 (0–2)	0.779
**Fruits**			
Baseline	3 (1–5)	2 (1–4)	0.472
End line	5 (3–7)	4 (2–7)	0.422
**Red/orange fruits**			
Baseline	2 (0–3)	1 (1–3)	0.879
End line	2 (1–4)	2 (1–4)	0.317
**Other fruits**			
Baseline	1 (0–2)	0 (0–1)	0.114
End line	2 (1–3)	2 (1–4)	0.884
**Milk and milk products**			
Baseline	0 (0–3)	0 (0–1)	<0.001
End line	2 (0–6)	2 (0–5)	0.512
**Fortified biscuits**			
Baseline	0 (0–2)	0 (0–0)	<0.001
End line	3 (2–7)	3 (1–6)	0.256

^1^ Values are median (25th, 75th percentiles); ^2^ Mann–Whitney test; ^3^ in children < 24 months old only (intervention *n* = 73; comparison *n* = 88); *p* < 0.05 indicates statistical significance.

**Table 3:** Child morbidity at baseline and end line, by treatment group.

Characteristics	Intervention (N = 200)		Comparison (N = 215)		*p*-Value ^1^
	*n*	%	*n*	%	
**Diarrhea**					
Baseline	38	19.0	25	11.6	0.037
End line	20	10.0	20	9.3	0.810
**Fever**					
Baseline	115	57.5	107	49.8	0.115
End line	92	46.0	102	47.4	0.769
**Cough**					
Baseline	132	66.0	114	53.0	0.007
End line	92	46.0	130	60.5	0.003
**Runny nose**					
Baseline	153	76.5	127	59.1	<0.001
End line	130	65.0	139	64.7	0.941

^1^ Chi-square to test for independence to assess any significant difference between treatment groups. *p* < 0.05 indicates statistical significance.

**Table 4:** Nutritional status at baseline and end line, by age and treatment groups ^1^.

Characteristics		Baseline			Endline		
	N	Intervention (N = 200)	Control (N = 215)	*p*-Value ^2^	Intervention (N = 200)	Control (N = 215)	*p*-Value ^4^
**WAZ**							
6–23 months	170	−0.94 ± 1.01	−1.02 ± 1.07	0.509 ^3^	−1.37 ± 0.86	−1.32 ± 1.06	0.047
≥24 months	245	−1.56 ± 0.90	−1.76 ± 0.76	0.112 ^3^	−1.39 ± 0.94	−1.58 ± 0.80	0.024
All	415	−1.32 ± 0.99	−1.45 ± 0.98	0.184 ^3^	−1.38 ± 0.90	−1.47 ± 0.93	0.535
**WHZ**							
6–23 months	170	−0.66 ± 1.01	−0.64 ± 1.07	0.537 ^3^	−0.89 ± 0.96	−0.84 ± 1.03	0.295
≥24 months	245	−0.88 ± 0.83	−0.99 ± 0.90	0.493 ^3^	−0.61 ± 0.96	−0.72 ± 0.97	0.474
All	415	−0.79 ± 0.91	−0.84 ± 0.99	0.620 ^3^	−0.72 ± 0.97	−0.77 ± 0.99	0.899
**HAZ**							
6–23 months	170	−0.49 (−1.43, 0.08)	−1.13 (−1.67, −0.25)	0.072	−1.36 (−1.88, −0.76)	−1.53 (−2.23, −1.01)	0.159
≥24 months	245	−1.75 (−2.34, −1.01)	−1.89 (−2.51, −1.44)	0.057	−1.75 (−2.28, −1.08)	−1.88 (−2.42, −1.38)	0.047
All	415	−1.42 (−2.14, −0.44)	−1.61 (−2.17, −0.95)	0.044	−1.58 (−2.23, −0.90)	−1.72 (−2.33, −1.23)	0.247
**Hemoglobin level**							
6–23 months	170	9.5 (8.5. 10.1)	9.3 (8.1. 10.0)	0.215	9.7 (8.8. 10.6)	9.7 (9.2 10.4)	0.538
≥24 months	245	10.6 (10.1. 11.3)	10.5 (9.8. 11.3)	0.412	11.1 (10.3. 11.7)	11 (10.1. 11.6)	0.286
All	415	10.3 (9.3. 11.0)	9.8 (9.0. 10.8)	0.071	10.8 (9.7. 11.4)	10.4 (9.6. 11.3)	0.617
**Underweight**							
6–23 months		12 (15.4)	20 (21.7)	0.291	19 (24.4)	25 (27.2)	0.751
≥24 months		40 (32.8)	45 (36.6)	0.532	35 (28.7)	40 (32.5)	0.704
All		52 (26)	65 (30.2)	0.338	54 (27)	65 (30.2)	0.900
**Wasting**							
6–23 months		10 (12.8)	13 (14.1)	0.803	9 (11.5)	12 (13)	0.837
≥24 months		9 (7.4)	16 (13)	0.981	9 (7.4)	9 (7.3)	0.566
All		19 (9.5)	29 (13.5)	0.204	18 (9)	21 (9.8)	0.746
**Stunted**							
6–23 months		9 (11.5)	12 (13)	0.766	18 (23.1)	27 (29.3)	0.607
≥24 months		46 (37.7)	57 (46.3)	0.171	43 (35.2)	52 (42.3)	0.136
All		55 (27.5)	69 (32.1)	0.307	61 (30.5)	79 (36.7)	0.103
**Anemia**							
6–23 months		71 (91)	90 (97.8)	0.048	64 (82.1)	81 (88)	0.272
≥24 months		77 (63.1)	78 (63.4)	0.961	55 (45.1)	60 (48.8)	0.562
All		148 (74)	168 (78.1)	0.323	119 (59.5)	141 (65.6)	0.134

^1^ Values are Mean ± SD or median (25th, 75th percentiles) or n (percentage); ^2^ Mann–Whitney test; ^3^ Independent *t*-test; ^4^ ANCOVA or logistic regression covariates: type of family (nuclear vs. extended), water source (pre-processed vs. natural source), house broken down after earthquake (Y/N), difficulties in accessing health service, education and place of worship (Y/N). *p* < 0.05 indicates statistical significance.

**Table 5:** Maternal stress (SRQ), psychosocial care (HOME) and developmental outcomes (BSID) at baseline and end line, by age and treatment groups ^1^.

Characteristics		Baseline			Endline		
	N	Intervention (N = 200)	Control (N = 215)	*p*-Value ^2^	Intervention (N = 200)	Control (N = 215)	*p*-Value ^3^
**SRQ Score**							
6–23 months	170	6 (4, 9)	5 (3, 7)	0.012	5 (2.75, 7)	5 (3, 8)	0.955
≥24 months	245	6 (4, 9)	5 (4, 8)	0.027	4 (3, 7)	4 (3, 8)	0.490
All	415	6 (4, 9)	5 (3, 7)	<0.001	5 (3, 7)	5 (3, 8)	0.546
**HOME Score**							
6–23 months	167	23 (21, 25)	24 (20, 27)	0.578	28 (24.2, 32)	27 (24, 31)	0.393
≥24 months	16	24 (21, 27)	24 (21, 27)	0.644	27 (26, 34)	28 (23.5, 32)	0.456
All	183	24 (21, 27)	24 (20, 27)	0.932	28 (25, 32)	27 (24, 31)	0.215
**BSID: adaptive behavior score**							
6–23 months	150				94.27 (88.55, 99.99)	99.74 (94.8, 104.99)	0.177
≥24 months	63				94.60 (84.41, 104.79)	86.01 (77.2, 94.78)	0.216
All	213				94.28 (89.21, 99.34)	95.59 (91.02, 100.16)	0.712
**BSID: cognitive score**							
6–23 months	152				97.19 (92.04, 102.33)	92.89 (88.09, 97.69)	0.242
≥24 months	64				95.61 (89.84, 101.38)	94.38 (89.32, 99.43)	0.754
All	216				96.66 (92.7, 100.59)	93.42 (89.82, 97.02)	0.242
**BSID: language score**							
6–23 months	152				85.73 (81.84, 89.63)	82.37 (78.74, 86.01)	0.228
≥24 months	64				93.42 (87.52, 99.32)	89.50 (84.33, 94.67)	0.333
All	216				87.76 (84.49, 91.02)	84.69 (81.70, 87.68)	0.183
**BSID: motoric score**							
6–23 months	152				92.41 (89.11, 95.70)	88.01 (84.93, 91.08)	0.063
≥24 months	64				97.50 (90.93, 104.07)	96.27 (90.51, 102.03)	0.784
All	216				93.82 (90.82, 96.82)	90.57 (87.82, 93.32)	0.124
**BSID: social emotional score**							
6–23 months	150				87.90 (83.76, 92.03)	90.73 (86.93, 94.53)	0.333
≥24 months	63				95.94 (89.51, 101.93)	85.26 (80.10, 90.42)	0.010
All	213				90.26 (86.82, 93.70)	88.97 (85.86, 92.07)	0.589

^1^ Values are median (25th, 75th percentiles) or n (percentage); ^2^ Mann–Whitney test; ^3^ ANCOVA or logistic regression covariates: type of family (nuclear vs. extended), water source (pre-processed vs. natural source), house broken down after earthquake (Y/N), difficulties in accessing health service, education and place of worship (Y/N). *p* < 0.05 indicates statistical significance. SRQ = self reported questionnaire score; HOME = home observation for the measurement of environment score; BSID = Bayley Infant Scale of Development.

## Data Availability

The data presented in this study are available on request from the corresponding author. The data are not publicly available due to privacy issue.
